# Registered nurses’ challenges and suggestions for improvement of their leadership close to older adults in municipal home healthcare

**DOI:** 10.1186/s12912-023-01215-x

**Published:** 2023-03-23

**Authors:** Erica Lillsjö, Kaisa Bjuresäter, Karin Josefsson

**Affiliations:** 1 Department of Health Sciences, Faculty of Health, Science, and Technology, Karlstad University, 651 88 Karlstad, Sweden; 2 Faculty for Nursing and Health Science, NORD University, 8026 Bodø, Norway

**Keywords:** Leadership, Registered nurse, Home healthcare, Older adult, Questionnaire, Municipal

## Abstract

**Background:**

Worldwide people are living longer. The need for healthcare for older adults is increasing. The trend is towards advanced home healthcare, where registered nurses are key figures. This implies challenges for municipal home healthcare, as well as for registered nurses’ leadership. The aim is to explore registered nurses’ perceptions of challenges and suggestions for improvements in their leadership close to older adults in municipal home healthcare.

**Methods:**

The present study is part of a larger web-based questionnaire survey with a cross-sectional design. The design is inductive, analysing data using qualitative content analysis and descriptive statistics. A questionnaire with open-ended and closed-ended questions was used. A total of *n* = 70 registered nurses leading close to older adults participated in seven municipalities in two geographic areas in Sweden.

**Results:**

The results show registered nurses’ perceptions of challenges as leaders close to older adults in terms of 11 categories. The categories are motivating for care, adjusting and coordinating nursing care to the older adult, relating to next of kin, managing communication difficulties, relating to social situations in the home, managing demands, working alone, having lack of time, collaborating with physicians, and care staff having low competence. The registered nurses suggested improvements for their leadership close to older adults in terms of nine categories. The categories are adjusting the work to the older adult, clarifying registered nurses’ responsibility, balancing demands and resources, setting time aside, improving staffs’ competence, ensuring staff’s competence development, improving the work environment, and cooperation between professions in the municipality, as well as between healthcare organizations.

**Conclusion:**

The results show that registered nurses’ leadership in municipal home healthcare implies a wide range of challenges. There is a need for strategies to improve the organizational preconditions to reduce challenges in registered nurses’ leadership in order to promote positive patient outcomes for safe and quality care.

## Background

Worldwide people are living longer [1], including in Sweden [2]. Therefore, the need for healthcare is expected to increase [1–3]. In Sweden, the responsibility for the care of older adults, 65 years and over, largely rests with Sweden’s municipalities [2, 4]. The number of older adults receiving care in ordinary housing is increasing [2]. This implies challenges for municipal home healthcare [2]. It is also challenging for registered nurses (RNs) to maintain patient safety when performing care in older adults’ home [5]. RNs’ leadership in direct care close to older adults is complex and implies handling challenges [6], which this study explores.

Sweden is divided into 290 municipalities and 21 regions [7]. Most municipalities have responsibility for healthcare in ordinary housing, that is, home healthcare, excluding healthcare provided by physicians, which is the responsibility of the regions [8]. During 2020, 215,200 older adults received home healthcare in Sweden at some time [9]. Home healthcare includes medical interventions, rehabilitation, habilitation and nursing performed by licensed healthcare professionals and care staff with delegation to perform tasks [10]. RNs have the overall nursing responsibility in home healthcare [10]. They cooperate with other professions such as occupational therapists, physiotherapists, physicians, care managers and care staff [11]. RNs delegate nursing care, such as the administration of medication to care staff, and are responsible for the task being performed, as well as performed in a correct way [12]. Delegation is necessary because RNs are responsible for a large number of older adults, making it impossible for RNs to complete all tasks by themselves [13].

Today, the trend is towards advanced care, which previously took place in hospitals [14], taking place in home healthcare [14, 15]. Patients are also discharged earlier from hospitals to home healthcare [16]. This means that more patients with increased complex healthcare needs are cared for in home healthcare [14, 16]. RNs in home healthcare work independently [16–18]. Thus, RNs requires broad competence in order to be able to handle unpredictable situations [18]. RNs’ competences include leadership [19], and they are responsible for leading patients’ nursing care to provide conditions for good and safe care [20].

There are many definitions of leadership [21]. Leadership differs from management [21, 22], although some of the processes of leadership can be found in management [21]. Management implies order and consistency, planning to achieve goals, staff planning and budgeting [22], while leadership can be described as a process to influence a group people to reach common goals [21]. Several different leadership styles have been described by Avolio et al. [23], focusing on relations, tasks and or situations, but none of them are directly related to the nursing context or nursing research. In the nursing literature, leadership is commonly described in terms of nursing leadership, healthcare leadership and clinical leadership [24]. Terms referring to leadership in nursing are used interchangeably and are not always used consistently, whether it refers to informal leadership or leadership linked to formal management [25]. A relation between leadership and patient outcome is described, such as leadership affects patient satisfaction [26], patient safety [26, 27] and quality of care [28].

In home healthcare, RNs has a leader role [6, 16]. RNs’ leadership includes ensuring customized and sufficient care by planning, supervising and following up the older adult’s care [16]. RNs coordinate the care for older adults [6, 14, 29] and are expected to guide care staff and solve problems [30]. RNs as leaders close to older adults, are described as being *multi-artists* [6], which entails trust and control, continuous learning, competence through knowledge and ability, nursing responsibility on an organizational level, application of skills, awareness of the individual’s needs and wholeness, mutual support, mutual relationships, collaborating on organizational and interpersonal levels, and exposure to challenges. Next of kin describe that RNs manage their leadership close to older adults despite lack of organizational preconditions [31]. Older adults describe RNs’ leadership close to them in home healthcare as valuable, coordinating the care they need [32].

To sum up, RNs’ leadership needs to be communicated as an important part of RNs’ professional role that contributes to a safe home healthcare for older adults [31, 32]. RNs have challenges in home healthcare, such as too extensive nursing responsibility [11], responsibility for patient safety [33], and palliative care [34], as well as information transfer between hospitals and home healthcare [35]. RNs describe lack of support and understanding of their complex work situation [36], and their leadership is often overlooked [37]. Support is needed in leadership for ultimately safe and quality care for positive patient outcomes [27]. RNs suggest improvements in home healthcare, such as clarifying the division of responsibilities between different professions [38]. However, research is sparse about RNs’ own perceptions of challenges specific to their leadership close to older adults. Therefore, the aim of this study is to explore RNs’ perceptions of challenges and suggestions for improvements in their leadership close to older adults in municipal home healthcare.

## Method

### Design

The present study is part of a larger questionnaire survey with a cross-sectional design [39] exploring RNs’ professional competence in municipal home healthcare for older adults. The data were collected in Sweden between May and November 2021. The design was inductive, analysing data using qualitative content analysis [40–42]. Those RNs of the RNs in the larger study who perceived challenges as a leader close to older adults and had suggestions for improvement for their leadership constitute the sample in the present study. The study was reviewed by the Health, Science, and Technology Faculty’s review of research ethics at Karlstad University (Dnr. HNT 2020/618).

### Setting and participants

The setting were seven municipalities in two geographic areas in Sweden. The target population in the larger survey was all RNs (*n* = 200) working as a leader close to older adults in home healthcare. RNs were included regardless of years working as an RN and having specialist education or not. Exclusion criteria were RNs working as managers far from the older adults. The total number of participating RNs in the larger survey was *n* = 71, comprising 36% of the target population.

The present study constitutes *n* = 70 (Table 1) and, out of these, RNs (*n* = 58) perceived challenges as a leader close to older adults, RNs (*n* = 51) stated that they talk to someone about their challenges, and RNs (*n* = 29) stated suggestions for improvement of their leadership close to older adults.

**Table 1:** Registered nurses’ (RNs) (*n* = 70) characteristics

Characteristics		
Female	*n* (%)	67 (96)
Age ^a^	Mean (SD) min-max	48 (11.6) 24–73
Year of nursing graduation ^a^	Median min-max	2006 1981–2020
Specialisation in nursing ^b^	*n* (%)	29 (43)
Employment		
Permanent	*n* (%)	68 (97)
Work full time	*n* (%)	44 (63)
Years worked		
As a RN ^c^	Mean (SD) min-max	16.4 (9.2) 1–38
At current work place ^c^	Mean (SD) min-max	6.7 (6.6) 3–35
As a RN caring for older adults ^d^	Mean (SD) min-max	11.8 (8.5) .3–35
As a RN in home healthcare for older adults ^c^	Mean (SD) min-max	7.2 (6.3) .4–29

Internal losses: a = 1, b = 2, c = 3, d = 11

### Questionnaire

The questionnaire in the larger study consisted of three sections: (1) Background variables, such as age and gender; (2) Nurse Professional Competence Scale Short Form (NPC Scale-SF) with 35 questions [43]; and (3) 21 questions, of which three questions were open-ended. Twenty questions in section three were about RNs’ leadership close to older adults in municipal home healthcare. These questions were created according to 10 themes of what RNs’ leadership implies close to older adults in municipal home healthcare, derived from a systematic review [6]. The questions were done in collaboration with a statistician. The last question in section three was about RNs’ suggestions for improvements and was modified from Andersson [44] to suit the context of this study. There was an opportunity for participants to add their own comments at the end of the questionnaire.

In order to test the clarity of the questions about leadership in section three, three RNs with experience in municipal care for older adults were interviewed. The interviews did not elicit any changes other than a spelling mistake that was corrected.

The present study is based on the three open-ended questions in section three. The first two questions were derived from that RNs’ leadership close to older adults in municipal home healthcare implies challenges [6]. The first question was, ‘Are you as a leader close to older adults exposed to challenges?’ and the response categories were yes and no. If participants answered yes, they were asked to state what kind of challenges they were exposed to. The second question was, ‘Do you talk to someone about the challenges in your leadership close to older adults?, and the response categories were yes and no. If participants answered yes, they were asked to state who they talked to. The third question asked for RNs’ suggestions to improve their leadership close to older adults, the next of kin and care staff in the municipal home healthcare.

### Data collection

A convenience sample was used [39]. After obtaining permission from managers for municipal home healthcare, the unit mangers were contacted and asked to send an e-mail to the RNs with an invitation to participate. The e-mail included an information letter explaining the aim of the study. The information included that participation was voluntary, that participants could withdraw their participation without explanation, that data would be kept confidential and that the RNs’ identity was protected. The e-mail included a link to the web-based questionnaire. RNs gave their informed consent in the web-based questionnaire, and the questionnaire could not be answered without the informed consent. Two e-mail reminders were sent.

### Data analysis

Questions with response categories ‘yes’ and ‘no’ are represented in absolute frequency (number = *n*) and relative frequency (%), as well as the question on who RNs talk to about challenges. The responses to the open-ended questions consisted of one to 160 words. The responses to each question were analysed separately using qualitative content analysis with an inductive approach [40–42]. The analysis started with reading the data in its entirety several times and continued with dividing the data into meaning units, consisting of words or sentences related through content and context [40]. The meaning units where condensed and coded, maintaining the content close to the original text [41]. Next, the codes were sorted by looking for similarities and differences and abstracting these into subcategories and categories. To strengthen trustworthiness [40], the coding process, subcategories and categories were discussed continuously during the analysis by the research team. This implies to move back and forward in the analysis process until consensus had been reached.

### Ethical considerations

Conventional research ethics principles, as formulated in the Declaration of Helsinki [45] and in Swedish ethics testing legislation [46], were followed, taking into account information, informed consent and confidentiality. RNs were informed of the aim of the study, that participation was voluntary, that they could withdraw their participation without explanation, that data would be kept confidential and that the RNs’ identity was protected. The results are described at a group level so that no individual RN can be identified.

## Results

The results describe RNs’ perceptions of challenges as a leader close to older adults in municipal home healthcare in terms of 11 categories (Table 2), as well as if, and who, RNs talk to about their challenges. The results also describe RNs’ suggestions for improvements in their leadership close to older adults in terms of nine categories (Table 3).

### Registered nurses’ perceptions of challenges as a leader close to older adults, and who they talk to about their challenges

The results show that *n =* 58 (83%) of the 70 RNs perceive that they are exposed to challenges as a leader close to older adults and 12 (17%) RNs answered that they were not. Of the RNs who answered that they were exposed to challenges (*n* = 58), 50 specified what type of challenges, which is presented in terms of 11 categories below (Table 2).

Of the 70 RNs, *n* = 51 (73%) had talked to someone about their challenges, *n* = 17 (24%) had not talked to someone and *n* = 2 (3%) did not answer the question. Of the RNs (*n* = 51) who had talked to someone, *n* = 44 specified who they talked to; their colleagues *n* = 37 (84%), other RNs *n* = 3 (7%), and managers *n* = 20 (45%), such as their closest manager and manager for home healthcare. Moreover, RNs (*n* = 44) talked about their challenges with care staff *n* = 4 (9%), physicians *n* = 2 (5%), medical responsible nurse *n* = 1 (2%), politicians *n* = 1 (2%), union *n* = 1 (2%) and occupational therapist *n* = 1 (2%). RNs also talked with the home care group *n* = 1 (2%), the total working group *n* = 1 (2%) and at team meetings *n* = 1 (2%).

**Table 2:** Overview of registered nurses’ (*n* = 50) perceptions of challenges in their leadership close to older adults

Sub-categories	Categories
Patients without insight	**Motivating for care**
Patients are unmotivated to receive care	
Patients do not want to receive care	
Next of kin do not want to receive care for their relative	
Provide care based on each individual’s wishes	**Adjusting nursing care to the older adult**
Adjust care to patients with dementia	
Several care providers involved in patient care	**Coordinating care for the older adult**
Quick discharges from hospitals	
Different journal systems	
Lack of material resources to coordinate patient visits	
Lack of conditions for teamwork	
Regulations	
Next of kin’s grief and sadness	**Relating to next of kin**
Next of kin at distance	
Deficient communication with patient due to disease	**Managing communication difficulties**
Older adult’s language difficulties	
Communication with next of kin	
Care staff’s language difficulties	
Deficient communication with staff	
Cultural differences	
Reach consensus in situations	
Deficient communication with another healthcare organizations	
Different social situations in the home	**Relating to social situations in the home**
Insufficient work environment at the older adult	
Perform correct assessments	**Managing demands**
Next of kin’s demands	
Care staff’s needs	
More advanced care and few registered nurses	
Lack of balance between demands and resources	
Work alone	**Working alone**
Not knowing what is going to happen	
Managers’ low support	
Difficult to keep up with all work tasks	**Having lack of time**
Too few staff	
Deficient availability of physicians	**Collaborating with physicians**
Deficient continuity of physicians	
Deficient responsibility of physicians	
Low competence affects quality of care	**Care staff having low competence**
Care staff lack competence	

#### Motivating for care

The results show that motivating older adults for care was a challenge for RNs as leaders close to older adults. It can be that older adults with nursing needs lack insight into their own best interests, such as when affected by diagnoses such as dementia. RNs perceived it as challenging that older adults can be unmotivated to receive care, such as due to poor compliance. Older adults may say no to prescribed care even if they are considered to have care needs. RNs also described it as challenging when next of kin opposed care to their relative.

*… it can sometimes be challenging when the patient does not understand their own best interests due to dementia. Difficult to lead and help in the best way …*.

#### Adjusting nursing care to the older adult

RNs perceive it as challenging as a leader close to older adults to provide care based on each individual’s wishes. Each individual has wishes regarding how they want to have it. Situations need to be solved with an open mind and in the best way based on each unique individual, their next of kin and their resources. RNs described that it is challenging as a leader to adjust care to older adults with dementia, such as medications, when patients have demanding behaviour and psychiatric symptoms.

*… everything is individual. It is important to be open-minded and open even to slightly different solutions, as long as it is good for the patient and his or her environment …*.

#### Coordinating care for the older adult

The results show that it is a challenge in RNs’ leadership when several care providers are involved in the older adult’s care. RNs described that it is complex when two healthcare organizations and different physicians are involved in the older adult’s care. RNs also perceive that a quick discharge from hospital is challenging. RNs described different journal systems as a challenge in their leadership; RNs have to spend time finding information to be able to provide safe care. This, RNs perceive, makes it difficult to reach a holistic view of the older adult’s care, and meet the needs of the older adult and the next of kin; this was described as a risk for patient safety.

RNs described the lack of material resources as challenging in their leadership, such as not having sufficient access to vehicles to be able to coordinate patient visits. RNs also perceived that the lack of conditions for teamwork and regulations was challenging in their leadership close to older adults.

*… we do not have access to the same journal system as the rest of the region, which makes the work more difficult and a lot of time is spent ‘hunting’ for information in order to provide safe care …*.

#### Relating to next of kin

RNs described that a challenge as a leader close to older adults may be handling the next of kin’s sadness and grief. RNs perceive that next of kin in grief are sometimes aggressive and questioning. It can be in situations involving next of kin’s sadness over their relative’s disease, or when their relative is in a palliative stage of their disease. RNs also perceive that it may be challenging when next of kin are at a distance, such as in another city or country.

*… in palliative care, next of kin’s grief can sometimes express itself through aggressiveness, or become strongly questioning …*.

#### Managing communication difficulties

The results show that communication difficulties with older adults, the next of kin and care staff may be a challenge for RNs as leaders close to older adults. RNs described that communication with the older adult can be deficient due to diseases, such as mental illness. The communication may also be affected when the older adult speaks a foreign language.

RNs perceive that care staff with language difficulties is a challenge in their leadership. Care staff who have difficulty writing and speaking the Swedish language affects communication with the older adults, such as in situations where the older adults have a hearing impairment or cognitive impairment. RNs also described that care staff’s language difficulties can affect the older adult’s care, such as leading to medication handling errors. RNs described deficiency in communication with staff, such as when care staff do not have a basic education, as a challenge for their leadership. RNs also perceive that cultural differences and reaching consensus in situations are a challenge. Deficiency in communication with other healthcare organizations is perceived as a challenge in RNs’ leadership.

*… many of the care staff have language problems, problems with reading and understanding Swedish, which affect the patient […] this often leads to medication handling errors …*.

#### Relating to social situations in the home

RNs perceive different social situations in the older adult’s home as a challenge for their leadership close to older adults, such as misery in the older adult’s home. The older adult’s finances may affect their ability to receive the nursing and medical care they need. RNs also described an insufficient work environment among some older adults as challenging.

*… I often see patients who have a great need for nursing care and need for social assistance; however, the patient’s finances have put an end to the patient getting the nursing care and medical care they really need …*.

#### Managing demands

The results show that a challenge for RNs as leaders close to older adults is managing different demands, both RNs’ own demands, as well as demands from others. RNs described that they constantly have to make choices and perform correct assessments, such as correct interventions and dealing with acute symptoms. RNs have to deal with the next of kin’s wishes and sometimes, as the RNs perceive it, unreasonable demands. RNs have to support care staff when the older adult’s care is influenced by different wishes and opinions. Care staff can make demands on the RNs to take action, such as when a patient’s condition deteriorates.

RNs described that there is a lack of balance between demands and resources; there are changes in home healthcare, with an increase in ill older adults and more need for advanced care and fewer RNs. RNs perceive that the number of older adults for whom an RN is responsible is too high and unreasonable.

*… absolutely challenges […] we now get much more ill patients to home healthcare but with the same conditions as before. More advanced care requires more registered nurses, and we have become fewer during the years I have worked in home healthcare …*.

#### Working alone

The results show that RNs perceive that working alone is a challenge in their leadership close to older adults. RNs described that being quite lonely in their work and not knowing what is going to happen are challenges for their leadership. Managers’ low support was also described by RNs as a challenge.

*… you are quite lonely in your work …*.

#### Having lack of time

RNs perceive the constant lack of time as challenging as a leader close to older adults. RNs described that lack of time affects their possibility to keep up with all work tasks, such as following prescriptions, working in teams, and leading and educating the care staff. Team meetings with care staff have to be de-prioritized, with the result that RNs meet the care staff infrequently. RNs perceived that the lack of time is related to too few staff. RNs described that understaffing affects the possibility to have a holistic view of the older adult’s care.

*… the conditions to lead, educate and work in teams do not exist. There is not enough time, the healthcare tasks come first …*.

#### Collaborating with physicians

RNs described that collaborating with physicians is a challenge in their leadership close to older adults. RNs perceived that physicians’ limited availability and time are challenging, such as when RNs need support with patient assessments. The deficiency of continuity of physicians in home healthcare was also a challenge, which, as RNs meant, may lead to that physicians ‘do not have knowledge’ about the older adults. RNs perceive that it is difficult to get physicians to take responsibility for older adults’ care.

*… difficulties to get in contact with physicians* w*hen there is a need for support in assessments …*.

#### Care staff having low competence

The results show that care staff lacking competence is a challenge for RNs as leaders close to older adults. RNs perceive that care staff’s low competence affects the older adult’s care, such as with care staff having difficulty following prescriptions. RNs described frustration over care staff’s low competence, which RNs perceived affects and creates suffering for the older adults. RNs described that care staff’s lack of knowledge leads to difficulties for RNs in doing their work.

*… the frustration over municipal care staffs’ low competence is great; it is also psychologically difficult for me to see that the patients suffer because of the low demands the employer has on the staff in the municipal care …*.

### Registered nurses’ suggestions for improvement in their leadership close to older adults

Of the 70 RNs, *n* = 29 left a written comment with suggestions for improvements in their leadership close to older adults; these are presented in terms of nine categories (Table 3).

**Table 3:** Overview of registered nurses’ (*n* = 29) suggestions for improvement of their leadership close to older adults

Sub-categories	Categories
Work according to person-centred care	**Adjusting the work to the older adult**
Increase staff continuity at the older adult	
Depart from detailed scheduling	
Illuminate healthcare provided in ordinary housing	**Clarifying the registered nurses’ responsibility**
Delimit registered nurse’s nursing responsibility	
Clarify registered nurse’s role in relation to managers for social care	
A sustainable number of patients within the registered nurse’s responsibility	**Balancing demands and resources**
Be able to deny care request when lacking registered nurses	
Improve staffing of registered nurses	
A balance between demands and resources	
Expand time for patient visits to increase patient safety	**Setting time aside**
Time for collegial support	
Time to lead for the registered nurse	
Time to delegate to care staff	
Registered nurses with experience	**Improving staff’s competence**
Language skills requirements for care staff	
Education requirements for care staff	
Increase competence of care staff to improve collaboration	
Improve instruments to validate knowledge and competence	
Education in leadership for registered nurses	**Ensuring staff’s competence development**
Education for care staff	
Education in conversation techniques	
Improve work environment for care staff	**Improving the work environment**
Improve communication between staff	**Improving the cooperation between professions in the municipality**
Increase team collaboration between professions	
Improve managers’ leadership	
Increase physicians’ availability in the municipality	
A common journal record system	**Improving cooperation between healthcare organizations**
Improve communication between healthcare organizations	
One head principal for health care	

#### Adjusting the work to the older adult

The RNs suggested, that to improve their leadership close to older adults, they should work in a systematic way according to person-centred care, and increase staff continuity for the older adults. The RNs also suggested to depart from detailed scheduling to improve their leadership. The RNs stated that the schedule for care staff is detailed and planned in minutes, which makes it difficult for RNs to lead and influence when and how the nursing should be conducted. If something unforeseen happens, there is no time to deal with it.

*… work more systematically with person-centred care …*.

#### Clarifying the registered nurse’s responsibility

The RNs suggested that healthcare provided by the municipality in ordinary housing needs to be illuminated. The RNs suggested, as an improvement in their leadership, that RNs’ nursing responsibilities in the municipalities should be delimited. The RNs perceived that their responsibility is medical healthcare; nursing was perceived as unlimited, and RNs cannot have overall responsibility for this. The RNs also suggested that their role has to be clarified in relation to managers for social care.

*… increased clarity around registered nurses’ role compared to unit manager for social care, for example for basic hygiene routines and general care …*

#### Balancing demands and resources

The RNs stated that there is a need for balance between demands and resources to improve their leadership and to be able to achieve quality of care. The number of older adults an RN is responsible for needs to be sustainable. The RNs suggested that it should be possible to deny care requests when there is a lack of RNs in the municipality. The RNs suggested that the staffing of RNs should be improved, with more RNs in home healthcare. This may lead to more time for each patient.

*… more balance between demands and resources is needed to provide good care. Otherwise, stress and conflicts between employees are created …*.

#### Setting time aside

The RNs described that they need increased time with the older adults to improve their leadership. The RNs perceived that increased time for visits to older adults would increase patient safety. The RNs suggested that time should be set aside for reflection, support and supervision with colleagues. The RNs also described that there has to be time for RNs to lead and that their leadership should be more valued. The RNs perceived that time for delegation to care staff should be expanded, made visible and include time for follow-up of the delegation.

*… time for leadership and that leadership is valued more …*.

#### Improving staff’s competence

The RNs suggested that, to improve their leadership, staff’s competence should be improved. RNs working in home healthcare should have experience and care staff’s competence should be improved. The RNs suggested language skills requirements for care staff, as well as other educational requirements. RNs perceive that the care provided today in home healthcare is more advanced. To be able to provide advanced home healthcare, care staff need to be educated, as well as be able to speak and understand Swedish. RNs have to be able to rely on care staff understanding, for example, a delegation. Higher competence among care staff could facilitate and increase collaboration between RNs and care staff. The RNs suggested improved instruments to validate knowledge and competence.

*… requirements on background and education of the care staff must be increased. The level of care is much more advanced today than just five years ago. It is not possible to provide advanced home healthcare with care staff who do not have sufficient knowledge …*

#### Ensuring staff’s competence development

The RNs stated that they need regular education in leadership. The RNs suggested that care staff also need time to be set aside for education, which gladly may be led by RNs. The RNs suggested education using conversation techniques, such as motivational interviewing.

*… the registered nurse needs to receive education in being a leader in nursing within municipal healthcare …*

#### Improving the work environment

The RNs suggested that what would facilitate an improvement in their leadership would be to improve the work environment for care staff.

*… the care staff must have a better working environment …*.

#### Improving the cooperation between professions in the municipality

The results show that RNs suggested improved communication between staff to improve their leadership; information has to reach all team members in the group. Communication techniques should be used, such as the situation, background, assessment and recommendation (SBAR). The RNs stated that there has to be better cooperation between different professions and increased team collaboration. The RNs suggested that managers’ leadership has to be improved, and dysfunctional managers need to be replaced. The RNs suggested that there is a need for the increased availability of physicians in the municipality, such as hiring physicians in the municipalities.

*… we need to go back to teamwork between different professions …*.

#### Improving cooperation between healthcare organizations

The results show that RNs suggested improved cooperation between healthcare organizations to improve their leadership close to older adults. The RNs suggested that a common journal system, improved communication between healthcare organizations and one head principal for healthcare could improve their leadership.

*… closer cooperation, increased accessibility for care staff and registered nurses to information between municipality and region …*.

## Discussion

RNs perceive a wide range of challenges in their leadership close to older adults. RNs perceive challenges in their leadership coordinating the older adult’s care, when different care providers are involved and getting information to be able to provide safe care. Safe care must be based on trusting relationships with patients, next of kin, colleagues and other staff [5]. A current study describes that RNs in home healthcare take responsibility for coordinating the care actors, which is described as a complex task [47]. Older adults have described this as important, and that they value RNs in home healthcare coordinating their care needs [32]. Claesson et al. [6] showed that RNs’ leadership close to older adults implies a complex leadership role, entailing RNs performing as *multi artists*. In the light of this and the results of this study, RNs’ leadership close to older adults in home healthcare entails a leadership role that is central to older adults receiving functioning care. The leadership implies that RNs are handling a wide range of challenges, although they do not always have the proper organizational preconditions [31].

Furthermore, this study shows that RNs perceive low support for their leadership from their managers. RNs need support from managers in the municipalities, as reported by earlier studies [48, 49]. RNs need support from managers to promote patient safety and cooperation among staff [48], although RNs have described that managers often fail to recognize RNs’ needs in their work [49]. Managers are not always familiar with RNs’ work in home healthcare, due to different educational backgrounds [48]. Managers’ support has been described as important for RNs to perceive themselves as leaders [50]. Therefore, in home healthcare, where RNs as leaders have an important and complex role that implies a wide range of challenges, they need managers’ support. Managers may support RNs’ leadership and promote RNs’ organizational preconditions to lead close to older adults.

RNs perceived collaborating with physicians as a challenge, because of issues with continuity and the availability of physicians, and because their responsibility is deficient. This is in line with earlier studies in which home healthcare providers reported substantial barriers in communication with physicians, such as trouble getting in touch with physicians [51]. Lack of cooperation concerning patients’ care leads to RNs in home healthcare feeling abandoned and alone in their caring work [52]. This also leads to an insecure healthcare situation for older adults [52]. Thus, strategies to improve communication between home healthcare providers, including physicians, must be developed.

The RNs in this study suggested improving their leadership by regularly receiving education on leadership. This result is in line with Sundberg et al. [50], who found that RNs leading in psychiatric care wanted more leadership training to be able to lead nursing care. RNs in municipalities are expected to have leadership competence [53, 54]. However, Josefsson and Hansson [19] reported that RNs in municipalities perceived a lack of competence development in leadership. Claesson et al. [6] stated that RNs must be prepared for their leadership in home healthcare during nursing education; at the same time, leadership has been described by newly graduated RNs as an ability which develops over time [55]. Kouzes and Posner [56] stated that leadership is not a characteristic of a specific personality, but rather that leadership is something that can be developed over time and learned. Therefore, regular education in leadership could be a possibility to strengthen RNs’ leadership in home healthcare. This may increase RNs’ ability to handle challenges in their leadership. To strengthen RNs’ leadership ought to be prioritized in relation to the expected challenges of home healthcare, which the present study also indicated. RNs in this study perceived several challenges as leaders, such as an increasing number of more ill older adults, and the need for more advanced care, results that are also in accordance with earlier studies [14–16, 48, 57].

The results show that lack of time is a challenge in RNs’ leadership, and RNs perceived this as affecting their possibility to perform all work tasks, follow prescriptions and work in teams. Lack of time as a challenge in home healthcare has been described in earlier research [48, 58–60]. Lack of time affects RNs’ possibility to keep up with new knowledge and research, despite the need for this due to increased advanced care, and new treatments and medical equipment [48]. Although, RNs in their profession are responsible for keeping up with new knowledge [20] and research to provide care based on evidence [20, 61]. Lack of time also affects RNs’ possibility to focus on more than practical tasks, such as being present, answering questions and caring for psychosocial needs [57]. RNs should also have a holistic view of the older adult’s care, including psychosocial, spiritual and cultural needs [20]. RNs’ leadership implies being able to identify the older adult’s needs and see the older adult in a holistic manner [6]. Lack of time has also been described as affecting patients in home healthcare, such as they avoid asking RNs questions [57]. Further research is required to be able to reach a deeper understanding of how RNs’ lack of time challenges their leadership and especially the leader style focusing on relations.

RNs in the present study perceive that lack of time affects their possibility to lead and educate care staff. Moreover, RNs perceive that care staff’s lack of competence is a challenge for their leadership. In Sweden, it has been described as necessary for RNs to delegate work tasks in nursing care to care staff to make home healthcare work [13, 30]; education is often included in the delegation [13]. A comparison between 2005 and 2015 shows that medical tasks performed by care staff, such as giving injections, had increased [62]. In the light of this, RNs’ lack of time to lead and educate care staff, combined with care staff’s lack of competence, may affect patient safety, leading to issues such as medication-handling errors. A current report by the Health and Social Care Inspectorate (IVO) [63] in Sweden illuminated that RNs in municipal nursing homes do not always feel secure that delegated medication administration is done in a patient-safe way. Moreover, 88% of RNs did not always have preconditions to support care staff. To follow up delegations and support care staff are highlighted as important by the IVO to be able to provide safe care [63]. RNs in the present study suggested improvement for their leadership to set time aside for delegation and to follow up delegations, as well as to improve care staff’s competence with requirements for proper education. These measures require a leader style focusing on both relations and tasks and should be prioritized to be able to provide safe home healthcare. In earlier research, the competence level of care staff were described as limited explored [54], in spite of their important role in municipal healthcare [54], often being front-line staff who report to RNs [64]. Therefore, further research is needed on how the competence level of care staff in home healthcare affects RNs’ leadership.

RNs in the current study perceive that care staff with difficulties in speaking and reading the Swedish language was a challenge in their leadership. This could lead to communication difficulties with the older adult and a risk of medication-handling errors. This is in line with the Health and Social Care Inspectorate report [63], which reported that 87% of RNs in municipal nursing homes perceived that there is a risk for patient safety due to care staff’s knowledge of the Swedish language. To speak and read the country’s native language is necessary to be able to understand instructions and delegations from RNs [63]. Care staff’s language difficulties in home healthcare has been, to our knowledge, sparsely explored and more research is needed. To not understand each other’s language ought to be challenging for RNs to lead regardless of leadership styles; focusing on relations, tasks and or situations.

The results of this study show that, RNs perceive challenges in their leadership to adjust care to older adults and provide care based on each individual’s needs and wishes. RNs suggested an improvement to their leadership by working more according to person-centred care. Person-centred care is an approach with the unique person in the centre. This approach needs to be applied systematically and consistently, with a patient-provider partnership [65]. The International Council of Nurses [61] states that RNs should provide person-centred care. Homecare providers describe it as stressful to balance the older adult’s needs with organizational demands [66]. Nevertheless, individualized care [67] and person-centred care [68] are described as core values in home healthcare [67, 68]. RNs’ leadership in home healthcare implies being able to adapt to the older adult’s needs and wishes [6]. Therefore, RNs in home healthcare need the proper organizational preconditions for their leadership to adjust and provide care in line with ethical guidelines for nursing to each older adult.

### Limitations

This study has some limitations. One might be that the answers to the open-ended questions were relatively short. However, this applies to most questionnaires, and the choice of open-ended questions for data collection gave RNs an opportunity to answer in their own words to get a fuller perspective of their perceptions [39]. The lack of an opportunity to ask follow-up questions to clarify the RNs’ answers might also be a weakness. Although this applies to most questionnaires, the use of a questionnaire in which the participants only answered questions in those domains determined by the researcher might be a weakness. On the other hand, participants were given an opportunity to answer through open-ended questions and to comment at the end of the questionnaire. Consequently, the transferability of the results is limited, since the sample was not chosen randomly. The participants were also asked to participate by their unit managers, allowing for the possibility that not all RNs received an invitation, which could have affected the response rate negatively. However, the results may reflect the perceptions of RNs working in similar conditions, and the results have significant value and are relevant to clinical practice, and are thus useful in RNs’ leadership close to older adults in municipal home healthcare.

## Conclusion

The results show that RNs’ leadership close to older adults in municipal home healthcare implies a wide range of challenges. There is a need for strategies to improve the organizational preconditions to reduce the challenges in RNs’ leadership and to promote positive patient outcomes for safe and quality care.

## Data Availability

The datasets from this study are available from the corresponding author on reasonable request.
