# Correction to: Registered nurses’ challenges and suggestions for improvement of their leadership close to older adults in municipal home healthcare

**DOI:** 10.1186/s12912-023-01266-0

**Published:** 2023-03-30

**Authors:** Erica Lillsjö, Kaisa Bjuresäter, Karin Josefsson

**Affiliations:** 1 Department of Health Sciences, Faculty of Health, Science, and Technology, Karlstad University, Karlstad, 651 88 Sweden; 2 Faculty for Nursing and Health Science, NORD University, Bodø, 8026 Norway

**Correction to: BMC Nurs 22, 80 (2023).**

10.1186/s12912-023-01215-x

Following publication of the original article [1], the authors reported an error in Table 1.

Due to a typesetting error, there are three values in the HTML version of Table 1 has been mistakenly presented, which caused the temporary discrepancy between the HTML version and the PDF.

In the Table 1 of the HTML version:

Values “3–35” and “.3–35” were wrong, the correct values should read “.3–35” and “.8–35”.

The value “c = 3” in the footnote of Table 1 was wrong, the correct value should read “c = 5”.

The original article [1] has been updated.
